# Study on the Fluorination Process of Sc_2_O_3_ by NH_4_HF_2_

**DOI:** 10.3390/ma16175984

**Published:** 2023-08-31

**Authors:** Zhi Li, Chengwei Zhan, Huan Yu, Xitao Wang, Shouqiu Tang, Jixue Zhou, Jianhua Wu

**Affiliations:** Shandong Provincial Key Laboratory of High Strength Lightweight Metallic Materials, Advanced Materials Institute, Qilu University of Technology (Shandong Academy of Sciences), Jinan 250014, China

**Keywords:** Sc_2_O_3_, (NH_4_)_3_ScF_6_, NH_4_ScF_4_, (NH_4_)_2_Sc_3_F_11_, ScF_3_

## Abstract

Research on rare-earth fluorides is of urgent and critical importance for the preparation and emerging applications of high-purity alloys. The fluorination of Sc_2_O_3_ by NH_4_HF_2_ to fabricate ScF_3_ is investigated. The effects of the fluorination temperature, time and mass ratio of reactant on the fluorination rate and fluoride are discussed in this work. The fluorination reaction was first confirmed using thermodynamic calculation. The thermal and mass stability of the fluorination process were analyzed by thermogravimetric and differential scanning calorimetric (TG-DSC). The as-obtained products at different fluorination temperatures were characterized by Powder X-ray diffraction (PXRD), scanning electron microscopy (SEM) and high-resolution transmission electron microscopy (HRTEM). The results indicated that the fluorination began at room temperature (RT) with the formation of (NH_4_)_3_ScF_6_. With the increase of temperature, the reaction proceeded sequentially through the formation of NH_4_ScF_4_, (NH_4_)_2_Sc_3_F_11_, and finally ScF_3_. The fluorination rate increased with the increase of fluorination temperature and holding time. ScF_3_ with a purity of 99.997 wt.% could be obtained by fluorination at 400 °C for 2 h.

## 1. Introduction

With the extensive applications of the Sc element in superconducting materials [1], as well as in automotive [2], aerospace, military [3], fluorescence [4] and solid oxide fuel batteries [5,6,7,8], the requirements for the quality of scandium or its alloys are also increasing. However, obtaining these metals is very difficult due to the high chemical activity of scandium. ScF_3_ is stabilized, non-hygroscopic, and slightly soluble in water and mineral acids [9,10,11], which constitute an important raw material for the preparation of scandium and aluminum-scandium alloy by electrolytic process [12] or metallothermic method [13].

The production technologies of ScF_3_ from Sc_2_O_3_ involves three main methods: wet, gas and solid-phase fluorination, respectively. The wet preparation method using aqueous hydrofluoric acid (HF) has no specific requirement for equipment, but the efficiency of fluorination is inefficient. Meanwhile, the product has a high content of ScOF, which limits ScF_3_ applications in high-end fields [14]. Fluorination with hydrogen fluoride gas (HF) or fluorine gas (F_2_) is a relatively short-term procedure, but these are corrosive and poisonous gases. Solid-phase fluorination using ammonium fluoride (NH_4_F) or ammonium hydrogen fluoride (NH_4_HF_2_) [15] is an economical and efficient preparation method. NH_4_F is highly hygroscopic, which will lead to the oxygen contamination of fluoride. NH_4_HF_2_ is a widely used fluorinating medium at lower temperatures, the fluorination process carries fewer impurities, and the requirements for equipment are lower. At room temperature, NH_4_HF_2_ does not represent any significant environmental danger, since it is solid with a very low partial pressure, whereas, when heated, it becomes a powerful fluorinating reagent [16,17]. Therefore, NH_4_HF_2_ is widely used to purify minerals and prepare fluorides from rare-earth oxides, for example, silica concentrate desilication [18], metal slag desiliconization [19], desilication of zirconium concentrates [20] and the production of GdF_3_ [21], PuF_3_ [22], and YF_3_ [23].

It is reported that the fluorination process of metal oxides includes the multi-step decomposition of intermediate products, as shown in Table 1. Hao et al. [21] and Claux et al. [22] reported that GdF_3_ and PuF_3_ had been obtained through a two-step reaction, respectively. Mukherjee et al. [23] and Zhou et al. [24] suggested that the fluorination of metal oxides required three steps to complete. However, the fluorination processes of BeO_2_ [25], Al_2_O_3_ [26], and Fe_2_O_3_ [27] occur through three ammonium metal fluorides, respectively.

The reports on the preparation process of ScF_3_ by Sc_2_O_3_ and NH_4_HF_2_ are few. Meanwhile, there are also different opinions about the reaction mechanism of fluorination. Zhang et al. [28] pointed out that Sc_2_O_3_ and NH_4_HF_2_ underwent a pre-fluorination reaction during the mixing process to generate the intermediate product (NH_4_)_3_ScF_6_, which was thermally decomposed at 274.82 °C to form ScF_3_. The unreacted NH_4_HF_2_ in the pre-fluorination process was thermally decomposed in the fluorination furnace to form NH_3_ and HF, while Sc_2_O_3_ was fluorinated with HF to generate ScF_3_. Rakov et al. [29] concluded that the pre-fluorination product of (NH_4_)_3_ScF_6_ decomposed to NH_4_ScF_4_ at 260–290 °C and that NH_4_ScF_4_ decomposed to ScF_3_ at 340–350 °C. Sokolova et al. [30] pointed out that adding NaF to the aqueous solution of Sc_2_O_3_ and NH_4_HF_2_ will form Na(NH_4_)_2_ScF_6_/Na_3_ScF_6_. After treatment, ScF_3_ could be obtained.

The goal of this work is to explore the fluorination process of Sc_2_O_3_ and NH_4_HF_2_. The thermodynamic process of the fluorination reaction is predicated on theoretical calculation. The fluorination intermediate products are investigated. The results indicated that the fluorination started at room temperature (RT) and through the intermediate products of (NH_4_)_3_ScF_6_, NH_4_ScF_4_, (NH_4_)_2_Sc_3_F_11_ on to the final ScF_3_. The fluorination process of Sc_2_O_3_ and the decomposition process of fluorides have been analyzed to provide a new idea for how to obtain high-purity ScF_3_.

## 2. Materials and Methods

### 2.1. Materials

**Figure 1:**
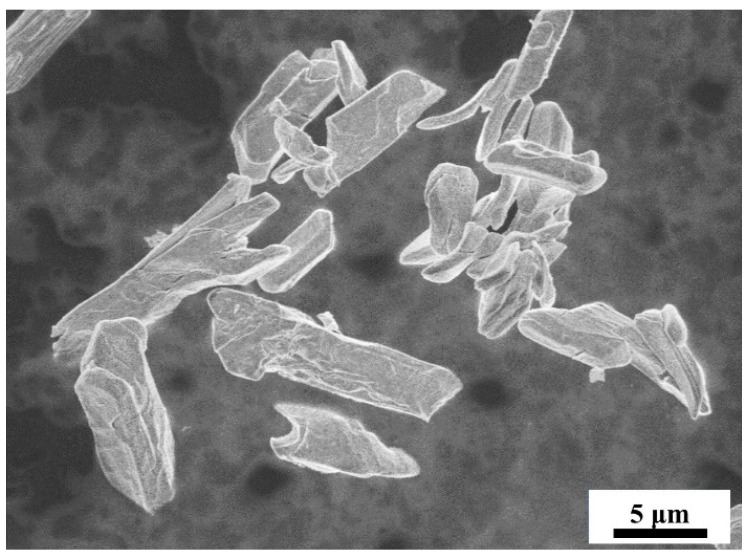
Microscopic morphology of Sc_2_O_3_ powders.

In this study, Sc_2_O_3_ powders (>99.9 wt.% purity) were fabricated by Hunan Oriental Scandium Industry, Hunan, China, as shown in Figure 1. Commercially available analytical reagent NH_4_HF_2_ crystals (Aladdin, Shanghai, China, >98.00 wt.% purity) were used as the fluorination reagent.

### 2.2. Preparation

**Figure 2:**
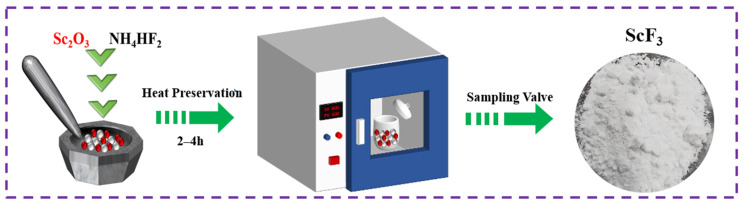
Schematic diagram of the fluorination experiment.

The mixtures (mass ratio of NH_4_HF_2_:Sc_2_O_3_ = 2 or 3) were ground and stirred in an alumina crucible for several minutes. Then, the mixtures were compacted to achieve full contact between Sc_2_O_3_ and NH_4_HF_2_ particles to promote the fluorination reaction. Subsequently, the mixtures were kept isothermal with different temperatures and times, and afterwards they were quickly cooled down to room temperature. To analyze the decomposition process of the fluorides, the mixture of Sc_2_O_3_ and NH_4_HF_2_ with a mass ratio of 1:3 was held at 100 °C for 24 h. The schematic diagram of the experimental process is shown in Figure 2.

### 2.3. Characterization

The powder X-ray diffraction (PXRD) of the samples was detected on a SmartLab 9 kW diffractometer (Rigaku, Tokyo, Japan) with Cu Ka radiation at a scanning rate of 10°/min under tube conditions of 40 KV and 60 mA. Thermogravimetric analysis and differential scanning calorimetry (TG-DSC) for a few mixtures of Sc_2_O_3_ and NH_4_HF_2_ were carried out on a TGA/DSC thermal analyzer (Mettler Toledo, Zurich, Switzerland) under argon atmosphere with a heating rate of 3 °C/min from RT to 500 °C in an alumina crucible. The microstructure and morphology were observed using a EVOMA 10 scanning electron microscope (SEM, Carl Zeiss AG, Oberkochen, Germany) with an energy dispersive spectrometer (EDS) to identify different elements. High-resolution transmission electron microscopy (HRTEM) and selected-area electron diffraction (SAED) images were performed on a Talos F200X transmission electron microscope (TEM, Thermo-Fisher Scientific, Waltham, MA, USA) with an accelerating voltage of 200 KV. The composition of the obtained ScF_3_ was identified using a iCAP 7400 inductively coupled plasma spectrometer (ICP, Thermo-Fisher Scientific, Waltham, MA, USA).

## 3. Results and Discussion

### 3.1. Thermodynamic Analysis

At a certain temperature, the value of the Gibbs free energy is a key factor, determining whether a reaction can proceed. The increase or decrease of $∆H^{0}$ can be a judgment dependent on the variation of the overall energy of the reaction. The thermal equilibrium constant K is used to indicate the reactivity of a chemical reaction at a specified temperature. A higher K value means that the reaction can be carried out thoroughly [31,32,33].

**Figure 3:**
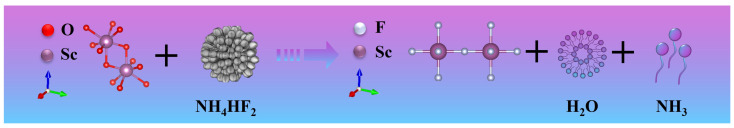
Schematic diagram of the fluorination reaction.

According to the previous introduction, the intermediate products will be formed during the fluorination process between NH_4_HF_2_ and Sc_2_O_3_. Due to the lack of thermodynamic data, this work does not discuss intermediate reactions. Thermodynamic data are only calculated when the final product is ScF_3_. Figure 3 shows the schematic diagram of the fluorination reaction process. It can be found that the rare-earth sesquioxides cubic structure of Sc_2_O_3_ turns into the cubic structure of ScF_3_ [34]. At the same time, NH_3_ and H_2_O are formed. The chemical equation may be expressed by Equation (1).

(1) $${Sc}_{2}O_{3}+3NH_{4}HF_{2}=2ScF_{3}+3NH_{3}\left(g\right)+3H_{2}O\left(g\right)$$

**Figure 4:**
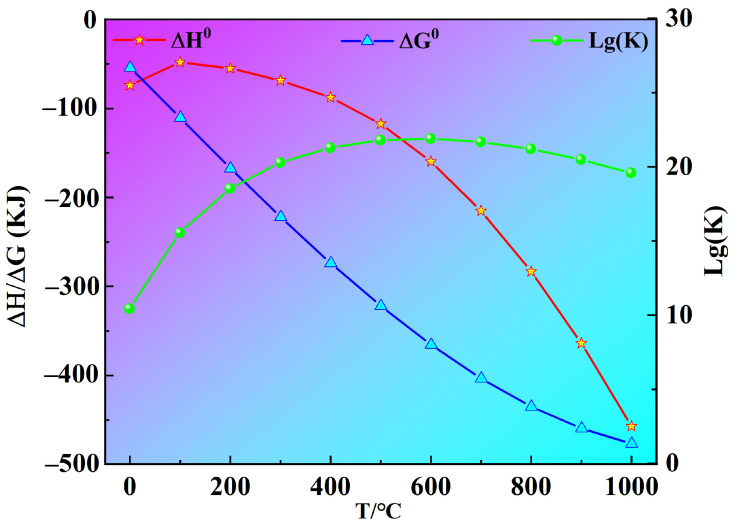
$∆H^{0}$, $∆G^{0}$ and Lg(K) versus reaction temperature, respectively.

The thermodynamic data of the fluorination reaction are calculated by HSC Chemistry 6.0 software, as shown in Table 2 and Figure 4. It is clear that $∆G^{0}$ < 0 in the temperature range 0–1000 °C, indicating that the reaction can proceed spontaneously. $∆H^{0}$ < 0 in the temperature range of 0–1000 °C, suggesting that the reaction of Sc_2_O_3_ and NH_4_HF_2_ is exothermic. Generally, the reaction equilibrium constant K > 10^5^ signifies that the reaction can occur thoroughly. Here, the Lg(K) is more than 9 under 0–1000 °C, confirming that the reaction can be carried out adequately. The results indicate that the fluorination reaction can easily occur under general conditions.

### 3.2. Fluorination Process Analysis 

**Figure 5:**
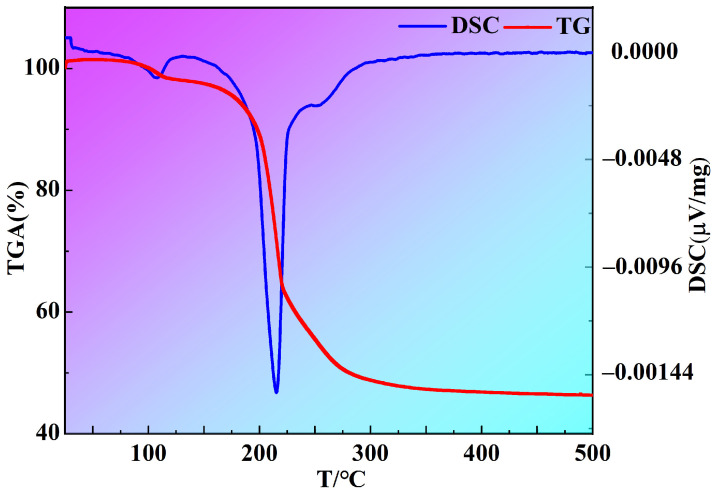
TG-DSC analysis of Sc_2_O_3_ and NH_4_HF_2_ mixtures with mass ratio of 1:3 after being held at 100 °C for 24 h.

Figure 5 shows the TG-DSC analysis of the Sc_2_O_3_ and NH_4_HF_2_ mixtures with a mass ratio of 1:3 after incubation in the 25–500 °C range. The mixtures were kept isothermal at 100 °C for 24 h to ensure that all Sc_2_O_3_ was converted into fluoride, while removing unreacted NH_4_HF_2_ and formed H_2_O [23,26,29]. The results show that there are three mass loss peaks at 120 °C, 220 °C and 260 °C and that they are 35%, 7%, and 10%, respectively. The total mass loss is approximately 52% during the fluoridation processes.

**Figure 6:**
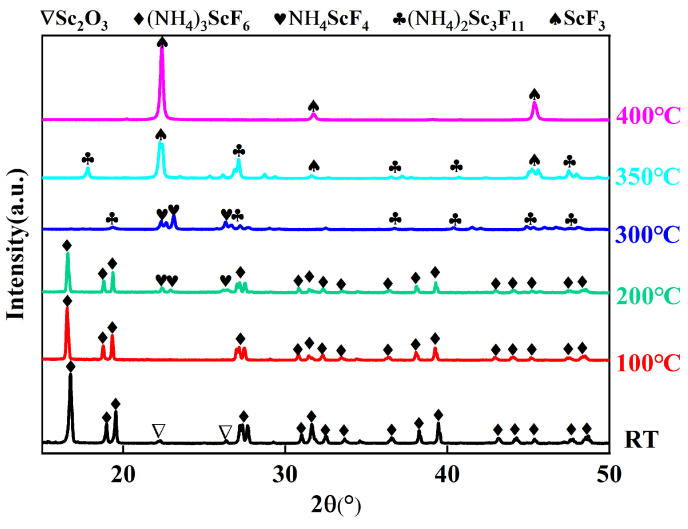
PXRD patterns of Sc_2_O_3_ and NH_4_HF_2_ mixtures with mass ratio of 1:3 after fluorination at different temperatures for 2 h.

The results of TG-DSC indicated that the thermal and mass changes were due to chemical reactions. To further identify and analyze the fluorides obtained in each of the reaction stages on TG-DSC curves, samples were prepared with different fluorination temperatures of RT, 100 °C, 200 °C, 300 °C, 350 °C, and 400 °C and held for 2 h, before being analyzed using PXRD, as shown in Figure 6. One can clearly see that the PXRD diffraction peaks of these fluorinated products occur without the diffraction patterns of ScOF.

The diffraction peaks of (NH_4_)_3_ScF_6_ (#97-001-9073) and Sc_2_O_3_ are detected in the PXRD of fluoride at room temperature. An interesting phenomenon was discovered, which was that the alumina crucible became hot during the mixing of the powders at room temperature. At the same time, the mixed powder gradually became moist. The results indicate that the fluorination of Sc_2_O_3_ by NH_4_HF_2_ had begun at room temperature, forming (NH_4_)_3_ScF_6_ and H_2_O by exothermic reaction. The possible reaction processes for the (NH_4_)_3_ScF_6_ at room temperature is shown in Equation (2). This is consistent with the results reported by Sokolova et al. [35]. Mukherjee et al. [23] also reported that fluorination of Y_2_O_3_ by NH_4_HF_2_ begins at room temperature with the formation of (NH_4_)_3_Y_2_F_9_.

(2) $${Sc}_{2}O_{3}+6NH_{4}HF_{2}=2{\left(NH_{4}\right)}_{3}ScF_{6}+3H_{2}O$$

When the fluorination temperature is 100 °C, only the diffraction peaks of (NH_4_)_3_ScF_6_ are observed. This illustrates that Sc_2_O_3_ had completely reacted and formed (NH_4_)_3_ScF_6_. This is due to the fact that the enhanced atomic activity with a rising temperature promoted the fluorination reaction.

As the fluorination temperature rises to 200 °C, diffraction peaks of NH_4_ScF_4_ (#97-024-0472) and (NH_4_)_3_ScF_6_ are detected. This indicates that (NH_4_)_3_ScF_6_ is gradually converted into NH_4_ScF_4_. The reaction can be expressed in Equation (3), which is a decomposition reaction. The theoretical calculation suggests that the mass loss before and after the reaction is 34.74%, which coincides with the 35% mass loss of the first stage in the TG curve.

(3) $${\left(NH_{4}\right)}_{3}ScF_{6}={NH}_{4}ScF_{4}+2NH_{3}\left(g\right)+2HF\left(g\right)$$

At a fluorination temperature of 300 °C, the diffraction peaks of (NH_4_)_3_ScF_6_ disappear from the PXRD pattern with the presence of diffraction peaks of NH_4_ScF_4_ and a new phase of (NH_4_)_2_Sc_3_F_11_ (#97-016-5543). This implies that (NH_4_)_3_ScF_6_ had been completely decomposed to NH_4_ScF_4_ at 300 °C and that NH_4_ScF_4_ had begun to transform into (NH_4_)_2_Sc_3_F_11_. The decomposition reaction of NH_4_ScF_4_ could be illustrated in Equation (4). The theoretical calculation reveals that the mass loss before and after the reaction is 8.79%, which corresponds to the 7% mass loss of the second stage in the TG curve.

(4) $$3{NH}_{4}ScF_{4}={\left(NH_{4}\right)}_{2}{Sc}_{3}F_{11}+NH_{3}\left(g\right)+HF\left(g\right)$$

The diffraction peaks of NH_4_ScF_4_ vanish from the PXRD pattern at 350 °C. Simultaneously, the diffraction peaks of the other phase of ScF_3_ (#97-007-7071) begin to appear. This is due to the fact that NH_4_ScF_4_ had thoroughly decomposed to (NH_4_)_2_Sc_3_F_11_, while (NH_4_)_2_Sc_3_F_11_ had gradually disintegrated to form ScF_3_. The decomposition reaction of (NH_4_)_2_Sc_3_F_11_ is shown in Equation (5). The theoretical calculation shows that the mass loss of the decomposition reaction is 11.58%, which is consistent with the 10% mass loss of the third stage in the TG curve.

(5) $${\left(NH_{4}\right)}_{2}{Sc}_{3}F_{11}=3ScF_{3}+2NH_{3}\left(g\right)+2HF\left(g\right)$$

All the diffraction peaks of ScF_3_ agree with the JCPDS card no. 97-007-7071, and no impurity is observed. This means that pure-phase samples with a crystal structure of ScF_3_ have been obtained at the fluorination temperature of 400 °C.

The above results show that (NH_4_)_3_ScF_6_ loses two NH_3_ and two HF to form NH_4_ScF_4_ at about 200 °C. NH_4_ScF_4_ will decompose to form (NH_4_)_2_Sc_3_F_11_, NH_3_ and HF at around 300 °C. At approximately 350 °C, (NH_4_)_2_Sc_3_F_11_ will decompose to form ScF_3_. In the practical production process, a rational and convenient preparation technology should be considered. Therefore, pure ScF_3_ can be obtained by fluorination of Sc_2_O_3_ with NH_4_HF_2_ at 400 °C for 2 h.

### 3.3. Microscopic Morphology and Structural Analysis 

**Figure 7:**
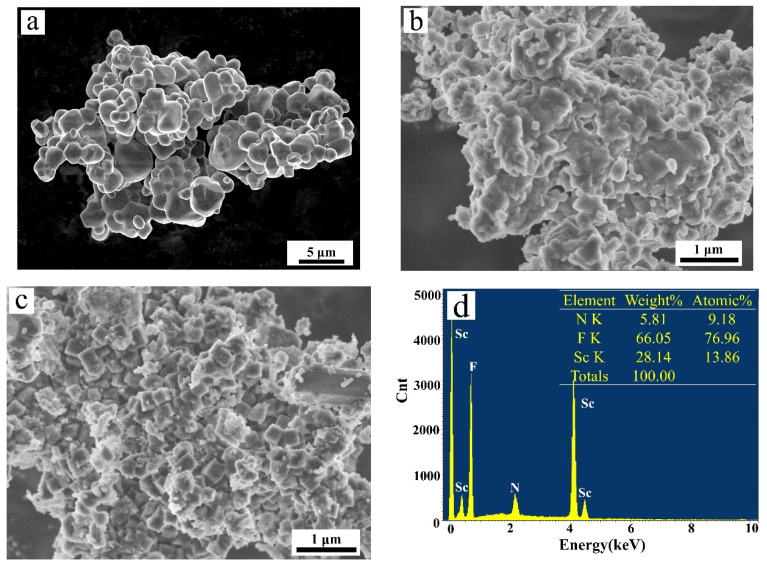

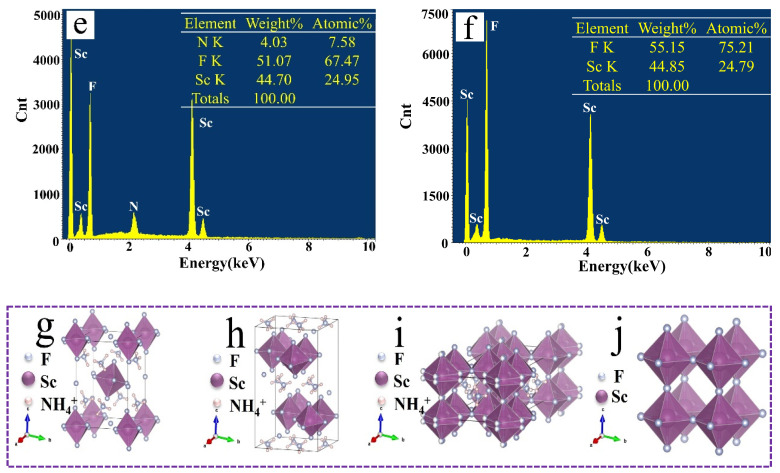
SEM images of (**a**) (NH_4_)_3_ScF_6_, (**b**) the mixture of NH_4_ScF_4_ and (NH_4_)_2_Sc_3_F_11_, and (**c**) ScF_3_. (**d**–**f**) EDS analysis. (**g**–**j**) Crystal structures of (NH_4_)_3_ScF_6_, NH_4_ScF_4_, (NH_4_)_2_Sc_3_F_11_ and ScF_3_.

Figure 7a–c shows the SEM images of (NH_4_)_3_ScF_6_, the mixture of NH_4_ScF_4_ and (NH_4_)_2_Sc_3_F_11_, and ScF_3_, respectively. It can be observed that the shapes of (NH_4_)_3_ScF_6_, NH_4_ScF_4_, and (NH_4_)_2_Sc_3_F_11_ are irregular, with varying average dimensions and morphologies. However, ScF_3_ mainly consists of a regular cubic structure with an average size of approximately 0.3 μm (Figure 7c), measured by Image-Pro Plus 6.0 image processing software. To obtain more detailed information about the content in the fluorides, the corresponding EDS analysis is exhibited in Figure 7d–f. Due to the large error in the measurement of light elements using EDS, we only pay attention to F and Sc elements here. It shows that the main components of fluorides are F and Sc and that the atom ratio F to Sc is about 6:1 in (NH_4_)_3_ScF_6_. Meanwhile, the atomic ratio F to Sc is close to 1:3 (Figure 7f), which is consistent with the atomic ratio of ScF_3_. Figure 7g–j illustrates the crystal structures of (NH_4_)_3_ScF_6_, NH_4_ScF_4_, (NH_4_)_2_Sc_3_F_11_ and ScF_3_. As shown in Figure 7j, ScF_3_ belongs to a cubic crystal structure, and the unit cell contains one Sc-atom (located at the center of octahedrons) and three F-atoms (located at the vertex of octahedrons).

**Figure 8:**
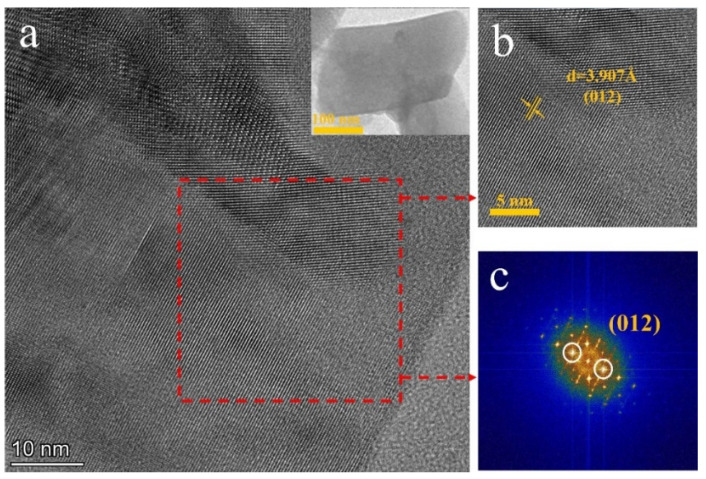
(**a**,**b**) HRTEM image and (**c**) SEAD pattern of (NH_4_)_3_ScF_6_ particle.

To further investigate the microstructures of (NH_4_)_3_ScF_6_ and ScF_3_, they were characterized by TEM. The HRTEM images of (NH_4_)_3_ScF_6_ particles are shown in Figure 8a,b. The inner-plane distance is about 3.907 Å, corresponding to the (012) crystal plane spacing of (NH_4_)_3_ScF_6_, which matches with the PXRD analysis of 3.835 Å in (NH_4_)_3_ScF_6_ (#97-001-9073). The SAED pattern of (NH_4_)_3_ScF_6_ is shown in Figure 8c, which confirms that (NH_4_)_3_ScF_6_ is a single-crystal structure with regular diffraction spots. Figure 7g shows the crystal structure model of (NH_4_)_3_ScF_6_, which has an octahedral chalcogenide structure as a result of the separated octahedral groups [ScF_6_]^3−^ and [NH_4_]^+^ [34,36,37,38]. The compound contains one site of Sc-ions, which occupy the 4a site and combine with six surrounding F-ions to form an octahedron [ScF_6_]^3−^ [38].

**Figure 9:**
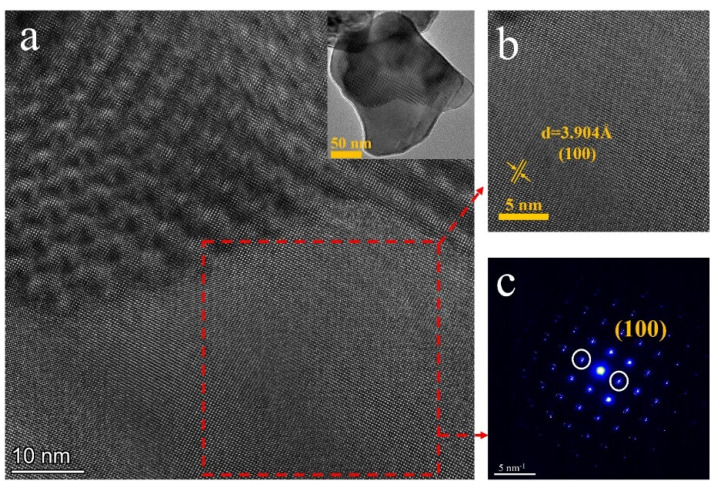
(**a**,**b**) HRTEM image and (**c**) SEAD pattern of ScF_3_ particle.

The HRTEM image and SAED pattern of the ScF_3_ particle are shown in Figure 9a–c. The results show that the interplanar space of about 3.904 Å corresponds to the (100) planes of ScF_3_, in keeping with the (100) crystalline spacing of 4.011 Å in ScF_3_ (#97-007-7071). The SAED was performed on a typical individual cube of ScF_3,_ as shown in Figure 9c. The sharp diffraction spots verify that ScF_3_ is well developed in a single-crystalline structure with a crystallographic orientation [11].

To further research the purity, the as-prepared ScF_3_ synthesized at 400 °C for 2 h was analyzed by ICP, as shown in Table 3. The results display that the purity of ScF_3_ reached 99.997 wt.%. This further proves that relatively pure ScF_3_ can be prepared by the fluorination of Sc_2_O_3_ with NH_4_HF_2_ at 400 °C for 2 h.

### 3.4. Effect of Temperature on Fluorination

The reaction temperature is an important factor affecting the fluorination process. In order to indicate the degree of fluorination reaction, the fluorination rate *k*% is defined as follows [33]:

(6) $$k\%=\frac{m_{F}}{M_{F}}\times 100\%$$

where $m_{F}$ is the theoretical mass of ScF_3_ after the fluorination, and $M_{F}$ is the mass of fluoride after the fluorination.

**Figure 10:**
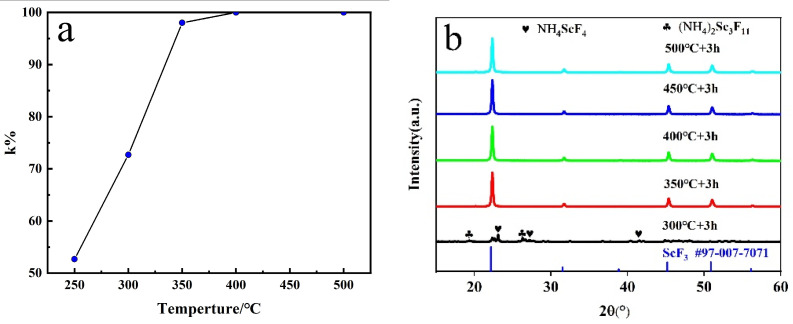
(**a**) Effect of temperature on fluorination rate, and (**b**) PXRD patterns of Sc_2_O_3_ and NH_4_HF_2_ mixtures with mass ratio of 1:3 after fluorination at different temperatures for 3 h.

Figure 10a shows the fluorination rate *k*% of ScF_3_ after the fluorination at different temperatures. In the range of 250–400 °C, *k*% is enhanced from 51.60% to 99.99% as the reaction temperature increases. The PXRD patterns of Sc_2_O_3_ and NH_4_HF_2_ mixtures with a mass ratio of 1:3 after fluorination at different temperatures for 3 h were detected, as shown in Figure 10b. Only the diffraction peaks of ScF_3_ (#97-007-7071) are observed when the fluorination temperature is above 350 °C. This proves that the reaction can be fully carried out at a lower temperature. The results indicate that the reaction can be carried out adequately with a rising temperature. This is consistent with the thermodynamic calculations.

### 3.5. Effect of Time on Fluorination

**Figure 11:**
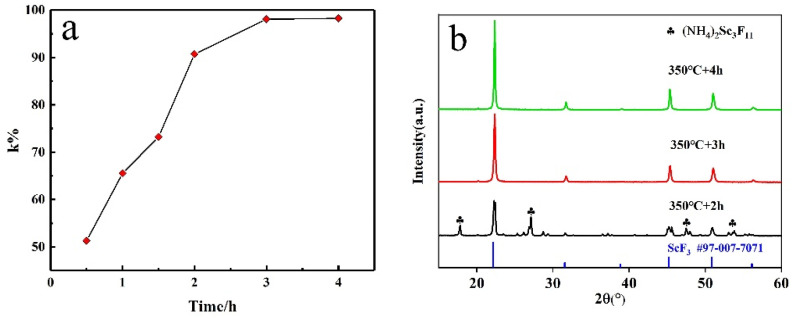
(**a**) Effect of holding time on fluorination rate at 350 °C. (**b**) PXRD patterns of Sc_2_O_3_ and NH_4_HF_2_ mixtures with mass ratio of 1:3 after fluorination at 350 °C for different times.

The holding time was also a significant influencing factor. The fluorination rate *k*% of ScF_3_ after fluorination at 350 °C for different times is shown in Figure 11a. The results show that the fluorination rate *k*% also increases with an increase in the holding time. The growth rate of *k*% at 0.5–2 h is significantly faster than at 2–4 h, indicating that the fluorination reaction has made great progress in the early stage. The fluorination rate *k*% can reach 98% when the fluorination time is 3 h. Figure 11b shows the PXRD patterns of fluoride at different holding times. The diffraction peaks of ScF3 (#97-007-7071) are observed; when the fluorination time is above 3 h, there are no diffraction peaks.

The above results reveal that the fluorination reaction can also be carried out relatively thoroughly at lower temperatures and with enough holding time.

### 3.6. Effect of Mass Ratio on Fluorination

**Figure 12:**
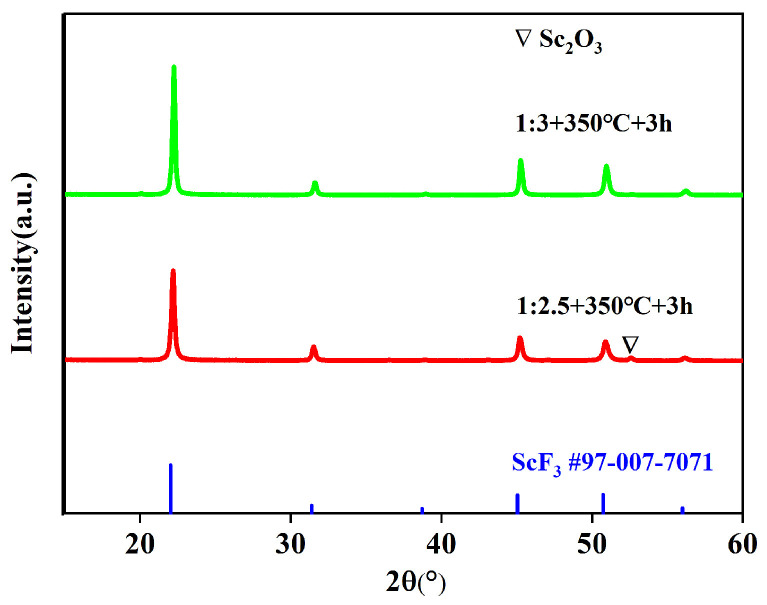
PXRD patterns of Sc_2_O_3_ and NH_4_HF_2_ mixtures with different mass ratios after fluorination at 350 °C for 3 h.

In addition to the fluorination temperature and time, the mass ratio of Sc_2_O_3_ to NH_4_HF_2_ also affects the fluorination results. According to the chemical Equation (2), the theoretical mass ratio of Sc_2_O_3_ and NH_4_HF_2_ is about 1:2.47. Two sets of samples were prepared with a mass ratio of 1:2.5 or 1:3 at 350 °C for 3 h. Figure 12 shows the PXRD patterns of the samples. It can be found that the mass ratios of Sc_2_O_3_/NH_4_HF_2_ can influence the final product. The diffraction peaks of ScF_3_ (#97-007-7071) and Sc_2_O_3_ are detected in the PXRD of the fluoride at a mass ratio of 1:2.5. This indicates that the fluoridation process is incomplete. Because the boiling point of NH_4_HF_2_ is about 230 °C, NH_4_HF_2_ could cause mass loss during the experimental process. This leads to a residue of Sc_2_O_3_. When the ratio is 1:3, only the diffraction peaks of ScF_3_ (#97-007-7071) are observed.

The chemical reaction is a process of reaching equilibrium. As the reaction progresses, the concentration of reactants gradually decreases, and the reaction rate slows down. The excess of raw materials helps the reaction move in the direction of generating products, which makes the reaction more complete and increases the reaction rate. Meanwhile, diffusion difficulties can hinder the development of the reaction. Therefore, the appropriate excess of NH_4_HF_2_ is beneficial for obtaining high-purity ScF_3_.

## 4. Conclusions

In this paper, high-quality ScF_3_ has been synthesized via a facile solid-phase fluorination method using Sc_2_O_3_ and NH_4_HF_2_. The fluorination process is investigated by TG-DSC and PXRD. The fluorination temperature has a critical impact on controlling the fluoride. The main conclusions are summed up below:

(1) The fluorination thermodynamic process between NH_4_HF_2_ and Sc_2_O_3_ is calculated. In the range of 0–1000 °C, $∆G^{0}$ < 0, $∆H^{0}$ < 0, and Lg(K) > 9, confirming that the fluorination is a spontaneous exothermic reaction and can be carried out completely.
(2) The fluorination of Sc_2_O_3_ by NH_4_HF_2_ begins at room temperature, forming (NH_4_)_3_ScF_6_ and H_2_O by exothermic reaction. As the temperature increases, a series of decomposition reactions will occur, so as to form: NH_4_ScF_4_→(NH_4_)_2_Sc_3_F_11_→ScF_3_. The PXRD diffraction peaks of the fluorides indicate that ScOF is not produced during the fluorination process.
(3) ScF_3_ with a purity of 99.997 wt.% can be obtained at 400 °C for 2 h. The ScF_3_ shows a regular cubic structure with an average size of 0.3 μm.
(4) The effects of the fluorination temperature, time, and mass ratio of raw materials were investigated. The results indicated that the fluorination rate increased with an increase of the reaction temperature and time. When the mass ratio of NH_4_HF_2_ to Sc_2_O_3_ ≥ 3, ScF_3_ with a higher purity can be obtained.

## Figures and Tables

**Table 1:** Fluorination processes of metal oxides.

Metal	Ref.	Reaction Process
Gd	[21]	Gd_2_O_3_→NH_4_GdF_4_→GdF_3_
Pu	[22]	PuO_2_→(NH_4_)_2_PuF_6_→PuF_3_
Y	[23]	Y_2_O_3_→(NH_4_)_3_Y_2_F_9_→NH_4_Y_2_F_7_→YF_3_
Zr	[24]	ZrO_2_→(NH_4_)_3_ZrF_7_→(NH_4_)_2_ZrF_6_→ZrF_4_
Be	[25]	BeO_2_→(NH_4_)_2_BeF_4_→NH_4_BeF_3_→NH_4_Be_2_F_5_→BeF_2_
Al	[26]	Al_2_O_3_→(NH_4_)_3_AlF_6_→NH_4_AlF_4_→(NH_4_)_0.69_AlF_3_→AlF_3_
Fe	[27]	Fe_2_O_3_→(NH_4_)_3_FeF_6_→NH_4_FeF_4_→(NH_4_)_0.18_FeF_3_→FeF_3_
Sc	[28]	Sc_2_O_3_→(NH_4_)_3_ScF_6_→ScF_3_ NH_4_HF_2_→NH_3_ + HF & Sc_2_O_3_ + HF→ScF_3_
	[29]	Sc_2_O_3_→(NH_4_)_3_ScF_6_→NH_4_ScF_4_→ScF_3_
	[30]	Sc_2_O_3_→(NH_4_)_3_ScF_6_→Na(NH_4_)_2_ScF_6_/Na_3_ScF_6_→ScF_3_

**Table 2:** Thermodynamic data calculated by HSC Chemistry.

T/°C	$∆H^{0}$/KJ	$∆G^{0}$/KJ	Lg(K)
0	−73.987	−54.607	10.443
100	−48.133	−111.107	15.554
200	−54..895	−167.843	18.531
300	−68.582	−222.500	20.279
400	−87.741	−274.296	21.286
500	−116.727	−322.508	21.791
600	−159.638	−365.997	21.897
700	−215.030	−403.872	21.680
800	−283.161	−435.414	21.195
900	−363.771	−460.031	20.485
1000	−456.935	−477.250	19.582

**Table 3:** The chemical composition of ScF_3_ was identified by ICP.

Element	Sc	F	Bal.
Content (wt.%)	44.117	55.880	0.003

## Data Availability

All data are available from the corresponding author on reasonable request.
